# Epigenetic Regulatory Mechanisms Induced by Resveratrol

**DOI:** 10.3390/nu9111201

**Published:** 2017-11-01

**Authors:** Guilherme Felipe Santos Fernandes, Gabriel Dalio Bernardes Silva, Aline Renata Pavan, Diego Eidy Chiba, Chung Man Chin, Jean Leandro Dos Santos

**Affiliations:** 1 School of Pharmaceutical Sciences, São Paulo State University (UNESP), 14800903 Araraquara, Brazil; guilhermefelipe@outlook.com (G.F.S.F.); gabriel.dalio@hotmail.com (G.D.B.S.); alinerenatapavan2004@yahoo.com.br (A.R.P.); chiba.diego@outlook.com (D.E.C.); chungmc@fcfar.unesp.br (C.M.C.); 2 Institute of Chemistry, São Paulo State University (UNESP), 14800060 Araraquara, Brazil

**Keywords:** resveratrol, epigenetic targets, histone deacetylase, SIRT1, DNA methyltransferase, histone acetyltransferase

## Abstract

Resveratrol (RVT) is one of the main natural compounds studied worldwide due to its potential therapeutic use in the treatment of many diseases, including cancer, diabetes, cardiovascular diseases, neurodegenerative diseases and metabolic disorders. Nevertheless, the mechanism of action of RVT in all of these conditions is not completely understood, as it can modify not only biochemical pathways but also epigenetic mechanisms. In this paper, we analyze the biological activities exhibited by RVT with a focus on the epigenetic mechanisms, especially those related to DNA methyltransferase (DNMT), histone deacetylase (HDAC) and lysine-specific demethylase-1 (LSD1).

## 1. Introduction

Resveratrol (RVT, 3,5,4′-trihydroxystilbene) is a natural polyphenol compound that was first isolated from the roots of the white hellebore *Veratrum grandiflorum O. Loes* in 1939 by Michio Takaoka [1]. Currently, this natural product can be found in a variety of plants, especially grape species, in response to fungal infections and ultraviolet radiation [2,3,4]. RVT is a stilbene derivative that can exist in two isomers, namely cis-(*Z*) or trans-(*E*), and the latter can undergo isomerization to the cis-(*Z*) form when exposed to ultraviolet irradiation [5]. Both isomers can be found in plants in the free-form or linked to carbohydrates [6]. This polyphenol gained worldwide attention due to its association with the “French paradox”, a phenomena that describes the lower mortality rate due to coronary heart disease in France compared to the rest of Europe and the U.S., even with a diet rich in saturated fats [7]. The greater consumption of wine in France, which has a high concentration of RVT, was suggested as a possible explanation for the “French paradox” [8]. In fact, several studies have reported that RVT exerts a cardioprotective effect [9,10,11]. Additionally, a number of reviews have described multiple biological activities of RVT and its potential in the treatment of several diseases, including cancer [12,13], diabetes [14,15], inflammatory diseases [16], metabolic disorders [17,18], neurological diseases [19,20] and increasing lifespan [21,22].

Some studies have described the potential intracellular molecular targets of this polyphenol such as NF-κB, PI3K/Akt signaling, mTOR signaling, MAPK signaling, cyclooxygenases, phosphodiesterases, estrogen receptors, microRNAs and several protein kinases [23,24,25]. All of these targets can modulate several biochemical pathways as they are involved in several important cellular processes, including inflammation, cell metabolism, cell cycle regulation, cell signaling and posttranslational modification [26]. Additionally, a growing number of works associate an involvement in epigenetic targets with the beneficial effects of RVT. The components of cellular epigenetic pathways regulate covalent modifications in histone proteins and DNA molecules, which may in turn influence gene expression and silencing and thereby modify cellular processes such as apoptosis, maintenance of stem cell pluripotency, X-chromosome inactivation and genomic imprinting, without affecting the DNA sequence [27]. The main epigenetic modifications are DNA methylation and reversible histone modifications [28]. In fact, such epigenetic modifications are the important regulators of gene transcription and have been linked to a large number of human diseases [29,30]. 

Therefore, this review aims to discuss the mechanisms by which RVT modulates important epigenetic targets in order to exemplify its function in the regulation of several diseases and metabolic disorders. Among its epigenetic targets, we will focus on the following enzymes: DNA methyltransferase (DNMT), histone deacetylase (HDAC) and lysine-specific demethylase-1 (LSD1) [31,32].

## 2. DNA Methyltransferase

Mammalian DNA methylation is accomplished by a complex interplay among a family of DNA methyltransferase enzymes, including DNMT1, DNMT3A, DNMT3B and DNMT3L [33,34]. DNMT1 is the most abundant and major maintenance methyltransferase that assures faithful propagation of existing DNA methylation patterns [35]. DNMT3A and DNMT3B are two related proteins that are encoded by distinct genes and function mainly as de novo methyltransferases that establish DNA methylation patterns during gametogenesis and early embryogenesis [35,36]. The related member, DNMT3-like (DNMT3L), is a catalytically-inactive member of the DNMT3 family that functions as a co-factor for DNMT3A during embryonic development and genomic imprinting in germ cells [37,38,39]. DNMT2 (also known as tRNA aspartic acid methyltransferase 1; TRDMT1) is of special interest because this enzyme is an RNA methyltransferase. It has been demonstrated that this enzyme methylates cytosine-38 in the anticodon loop of aspartic acid tRNA instead of DNA [40,41].

The catalytically-active DNMT1 and DNMT3 enzyme families share common features in their catalytic C-terminal domain; however, they differ in their N-terminal regulatory regions [42,43]. The C-terminal region comprises 500 amino acids, harbors the catalytic center of the enzyme and is highly conserved among prokaryotes and eukaryotes. The N-terminal multi-domain comprises 621 amino acids and is essential for discriminating between hemimethylated and unmethylated DNA. The differences in the N-terminal region of the DNMTs may largely account for their distinct activities and functions in the DNA methylation process [40,44]. 

DNA methylation by DNMTs plays an important role in several fundamental cellular processes, such as embryonic development and differentiation, DNA repair and maintaining genome stability and chromatin structure [45,46,47]. An aberrant or abnormal expression of DNMTs is associated with a variety of human diseases, including cancer [46,48], neurological disorders [49,50], immunological diseases [51] and genetic disorders [52,53,54,55,56]. Nevertheless, cancers with abnormal DNA methylation patterns are the most extensively described diseases in the literature [46,47,57,58]. Therefore, DNMTs are interesting and very promising therapeutic epigenetic targets, wherein specific inhibitors might regulate their activity and stop or even reverse aberrant cellular processes [43,59]. Several nutritional compounds, including apigenin, genistein, curcumin, sulforaphane, epigallocatechin gallate (EGCG) and RVT, have been described as regulators of cellular epigenetic events, such as DNMT activity, in a variety of cancers [60,61,62,63]. 

Tollefsbol and coworkers reported that 15 µM RVT was able to decrease DNMT enzymatic activity and mRNA levels of DNMT1, DNMT3A and DNMT3B in HCC1806 breast cancer cells. Interestingly, significant alterations in DNMT activity were not detected in MCF10A control cells even after 72 h of the treatment [64]. Subsequently, the same research group expanded their studies to MDA-MB-157 breast cancer cells, an estrogen receptor-α (ERα)-negative breast cancer cell line [65]. ERα-negative breast cancers are clinically more aggressive and normally do not respond to conventional hormone-directed therapies [66,67]. RVT was able to decrease DNMT enzymatic activity and alter overall DNA methylation patterns by decreasing 5-methylcytosine levels, which in turn, led to a significant reduction in DNA methylation. This decrease in methylation through epigenetic mechanisms led to the reactivation of and an increase in ERα expression, which could have facilitated conventional hormone-directed therapies, such as tamoxifen [65]. In another study, Romagnolo and coworkers used MCF-7 breast cancer cells and reported that RVT was able to reduce DNMT1 activity at the BRCA-1 promoter [68]. The *BRCA-1* gene encodes a tumor suppressor protein involved in the repair of DNA damage, and its downregulation is associated with sporadic breast cancers [69]. Thus, reduction in methylation at the *BRCA-1* gene led to an increase in BRCA-1 protein expression [68]. Similarly, Ralhan and coworkers demonstrated that RVT reduced DNMT transcript levels by quantitating mRNA for each of the three DNMTs in MCF-7 and MDA MB 231 human breast cancer cell lines. Further, Western blot analysis revealed that, compared to untreated cells, DNMT1 protein expression was reduced by 2–3-fold after cells were exposed to 10 µM RVT [70]. In vivo studies also demonstrated that RVT (25 mg/kg/day) decreases DNMT3b expression in a rodent model of estrogen-dependent mammary carcinoma. The authors observed a significant reduction in DNMT3b expression in tumor tissue compared to normal mammary tissue; however, no significant change in DNMT1 protein expression was observed [71]. In contrast, a study by Stefanska and coworkers showed an opposite effect of RVT in human breast cancer cell lines MCF10CA1h and MCF10CA1a. These authors observed an upregulation of DNMT3b activity after an exposure to 15 µM RVT that led to the hypermethylation of the *MAML2* gene and its silencing [72]. MAML2 is a coactivator of the oncogenic NOTCH signaling pathway, which in turn regulates several processes essential for tumorigenesis [73,74].

## 3. Histone Deacetylase

Histones (H3, H4, H2A, H2B and H1) are important proteins involved in all chromatin-mediated processes including transcription, replication and repair [75,76], and the modulation of the balance between the acetylated and deacetylated states of histones has a central role in the transcriptional regulation [77]. HDACs are a class of enzymes that remove acetyl groups from the ε-*N*-acetyl lysine amino acid on histone and non-histone proteins. HDACs are divided into five classes: class I comprises HDAC1, HDAC2, HDAC3 and HDAC8; class IIa comprises HDAC4, HDAC5, HDAC7 and HDAC9; class IIb comprises HDAC6 and HDAC10; class III comprises the sirtuins SIRT1–SIRT7; and class IV contains HDAC11. Classes I, IIa, IIb and IV are dependent on divalent metal ions for their catalytic activity, whereas the sirtuins are NAD^+^-dependent enzymes and are structurally and biochemically unrelated to the other HDAC classes [78]. Histone deacetylation is often associated with transcriptional repression and gene silencing [27]. In vitro and in vivo models have demonstrated that RVT may modulate HDACs activity and thereby regulate gene expression in cells [79,80,81]. The following sections will present the modulatory effects of RVT on HDACs and their influence on various diseases and metabolic disorders. 

### 3.1. Metabolic Disorders

RVT was described as a protective agent against several metabolic diseases and disorders mainly through the activation of SIRT1 and thereby regulating a number of important genes involved in cellular metabolic control. Lagouge and coworkers reported that RVT improved mitochondrial function in mice, as indicated by increased running time and greater oxygen consumption by muscle fibers. This beneficial effect was associated with the activation of the protein deacetylase, SIRT1, which induced peroxisome proliferator-activated receptor-γ coactivator (PGC-1α) activity by deacetylating multiple lysine sites. PGC-1α is a cofactor that plays an important role in mitochondrial biogenesis and functions. Importantly, this increase in mitochondrial activity was not observed when SIRT1 expression was disrupted [82]. In another study, Kemper and collaborators demonstrated that activation of SIRT1 by RVT led to a reduction in acetylated farnesoid X receptor (FXR) levels in a mouse model of metabolic diseases, thereby leading to its activation. FXR plays a critical role in the regulation of lipid and glucose metabolism, and the disruption of its genes is associated with metabolic diseases, including diabetes and hypercholesterolemia [83]. RVT was also reported to have an inhibitory effect against high glucose-induced ‘metabolic memory’ of endothelial senescence. ‘Metabolic memory’ is the term used when transient hyperglycemia can potentiate persistent diabetic vascular complications. It was demonstrated, using human umbilical vascular endothelial cells cultured in high glucose media, that RVT can activate SIRT1 and then modulate its downstream pathways, including p300, p53 and p21 [84].

The induction of SIRT1 by RVT has also been observed in several studies on diabetes. Yun and collaborators reported that RVT was able to reduce superoxide production and cellular oxidative stress in human monocytic (THP-1) cells cultured in hyperglycemic conditions by upregulating SIRT1. This beneficial effect was not observed when SIRT1 was inhibited using small interfering RNA (siRNA) [85]. Similarly, Xu and collaborators reported that RVT can activate SIRT1 and protect rat mesangial cells cultured in high glucose medium from hyperglycemia-induced oxidative damage to the mitochondria. The levels of reactive oxygen species (ROS) and mitochondrial superoxide were decreased after a treatment with RVT. Furthermore, these authors observed that RVT-induced ATP production and preserved mitochondrial DNA content, and SIRT1 blockade led to the loss of these beneficial effects [86]. In an obese and diabetic mouse model, RVT was able to normalize hyperglycemia and improve hyperinsulinemia after long-term intracerebroventricular infusion of RVT (79.2 ng/day). The authors hypothesized that the antidiabetic effect of RVT could be related to SIRT1 expression in the central nervous system (CNS). Indeed, acetylated lysine 379 levels in p53 in the brain, used as an SIRT1 activity marker, were reduced in RVT-treated animals, compared to the untreated group [87]. A recent study in Sprague–Dawley rats fed on a high-fructose diet and treated with RVT showed recovery of hyper-anxiety induced by the metabolic syndrome. These authors suggested that RVT was able to protect from both metabolic and anxiety disorders by activating SIRT1, 6 and 7, as high levels of mRNA of these enzymes were observed in the striatum of the animals [88].

RVT also plays an important role in modulating insulin secretion by activating SIRT1. Multiple studies have shown its beneficial effects in reversing insulin resistance in vivo. Vetterli and collaborators showed that RVT was able to trigger glucose-stimulated insulin secretion in both INS-1E cells (insulin secreting beta cell) and human islets. The activation of SIRT1 is thought to be the main target of RVT, as the overexpression of SIRT1 facilitates RVT effects on insulin secretion and conversely, SIRT1 inhibition leads to the loss of these benefits. RVT also helps increase the glycolytic flux that results in increased glucose oxidation, ATP generation and mitochondrial oxygen consumption [89]. In another work, Sun and collaborators demonstrated that RVT at a dose of 2.5 mg/kg/day was able to increase SIRT1 levels in mice fed a high-fat diet, which led to an improvement in insulin sensitivity. This enhancement in insulin sensitivity was associated with an increase in the deacetylase activity of SIRT1, which was required for the repression of the protein tyrosine phosphatase 1B (PTP1B) transcription at the chromatin level. PTP1B is a negative regulator of the insulin signaling pathway [90]. Similarly, a two-year RVT administration regimen (80 mg/day in the first year and 480 mg/day in the second year) in rhesus monkeys led to an increase in SIRT1 expression and an improvement in insulin sensitivity in visceral white adipose tissue. In addition, NF-κB activity and adipocyte size were also reduced. This beneficial effect was also confirmed in cultured 3T3-L1 adipocytes, wherein SIRT1 protein levels increased and NF-κB phosphorylation decreased [91]. Wu and collaborators also report that treatment with 10 μmol/L of RVT for 24 h resulted in a significant increase in insulin secretion from the islets isolated from the rats fed on a high-fat diet. This effect was associated with the activation of SIRT1 by RVT, after the inhibition or knockdown of SIRT1 suppressed insulin transcription [92]. 

RVT also plays a role in the attenuation of diabetic nephropathy by activating SIRT1. Long-term RVT treatment of diabetic Sprague–Dawley rats showed increased mRNA content and protein expression of SIRT1 in the kidneys. This beneficial effect led to an amelioration of diabetic nephropathy and was associated with enhanced autophagy in the kidneys related to the upregulation of autophagic proteins such as Atg7, Atg5 and LC3. Additionally, SIRT1 activation was responsible for hypoxia-induced autophagy mediated by hypoxia-inducible factor-1a (Hif1a). Hif1a overexpression led to the upregulation of the pro-autophagic Bcl2/adenovirus E1 V 19-kDa interacting protein 3 (Bnip3) that plays an important role in the induction of autophagy, which is a process responsible for removing protein aggregates and damaged or excess organelles to maintain intracellular homeostasis and cellular integrity [93]. Wen and collaborators have also demonstrated the beneficial effects of RVT in ameliorating diabetic nephropathy through activation of SIRT1. In a type-1 diabetic rat model, the activation of SIRT1 by RVT led to the modulation of kidney angiogenesis, mainly by suppressing vascular endothelial growth factor (VEGF) expression and secretion. It is noteworthy that SIRT1 knockdown led to the mitigation of RVT’s effects [94]. In another work, Wu and collaborators demonstrated that RVT was able to increase forkhead transcription factor O1 (FoxO1) activity mediated by SIRT1 activation in diabetic rat kidneys. The transcription factor FoxO1 plays a crucial role in cell metabolism, aging and oxidative stress resistance, and its upregulation has been associated with cellular protection against oxidative stress. Thus, the authors suggested that the improvement in diabetic nephropathy mediated by RVT may be related to increased expression of FoXO1 [95], if oxidative stress is implicated in the pathogenesis of diabetic nephropathy [96,97]. Similarly, Huang et al. showed the beneficial effects of RVT in ameliorating diabetic nephropathy in diabetic rats through the activation of SIRT1 and a consequent reduction in oxidative stress levels [98].

Many studies suggest that the activation of SIRT1 by RVT is associated with the modulation of lipid metabolism. Hou and collaborators report that RVT was able to increase AMP-activated protein kinase (AMPK) activity by activating SIRT1, which in turn led to a reduction in its downstream targets, such as ACC (acetyl-CoA carboxylase), FAS (fatty acid synthase) expression and lipid accumulation in human HepG2 hepatocytes exposed to high glucose [99]. Picard and co-workers demonstrated that the treatment of 3T3-L1 adipocytes with 50 or 100 µM RVT reduced their fat content mainly by lowering triglycerides and stimulating free fatty acid release. Again, SIRT1 activation has been suggested to be the main mechanism, as its knockdown led to a suppression of the observed beneficial effects [100]. RVT was also able to increase oxidized low-density lipoprotein (Ox-LDL) degradation and clearance through the autophagy-lysosome pathway in human umbilical vein endothelial cells. This effect was associated with SIRT1 activation that then led to an increase in cathepsin D protein levels, a lysosomal proteinase that is most important for LDL degradation [101]. Kurano and collaborators reported that HepG2 cells and mice treated with RVT showed increased levels of intra- and extra-cellular apolipoprotein M (apoM), along with intracellular sphingosine 1-phosphate (S1P) levels. S1P plays an important role in cardiovascular diseases and exerts several beneficial effects, including anti-apoptosis activity, anti-inflammatory activity, vasorelaxation and preservation of vascular permeability. The increased levels of S1P were related to apoM upregulation mediated by SIRT1 activation. The authors suggested that this effect may be partly responsible for the antiatherosclerotic properties of RVT [102]. In another work, Ajmo and coworkers reported the beneficial effects of RVT in countering alcoholic fatty liver in mice chronically fed ethanol. The activation of the SIRT1-AMPK signaling pathway was associated with this effect, which led to the suppression of the sterol regulatory element binding protein 1 (SREBP-1) and the activation of the peroxisome proliferator-activated receptor γ coactivator α (PGC-1α), two important components of free fatty acids’ metabolism in alcoholic liver steatosis [103].

Mitochondrial disorders, such as increased production of ROS, are frequently observed in diabetes and are involved in the disruption of cellular energy balance, activation of inflammatory processes and endothelial dysfunction. Ungvari and collaborators reported that RVT attenuates mitochondrial oxidative stress in coronary arterial endothelial cells exposed to high glucose to mimic diabetic conditions by reducing mitochondrial ROS production. This effect was also associated with the activation of SIRT1, and while its knockdown led to the suppression of beneficial effects, its overexpression mimicked the beneficial effects mediated by RVT [104]. RVT-induced activation of SIRT1 results in an increase in the mitochondrial biogenesis. For instance, Csiszar and collaborators reported that RVT induced mitochondrial biogenesis in cultured human coronary arterial endothelial cells by enhancing mitochondrial mass and mitochondrial DNA content, overexpressing proteins of the electron transport chain and inducing mitochondrial biogenesis factors, such as PGC-1α, nuclear respiratory factor-1 (NRF1) and mitochondrial transcription factor A (mtTFA). The knock-down of SIRT1 by small interfering RNA suppressed these effects. Additionally, type 2 diabetic mice subjected to chronic treatment with RVT also showed an increase in the mitochondrial biogenesis in the aorta [105]. In another report, the treatment of C2C12 myotube cells with RVT resulted in a significant increase in mitochondria mainly due to the greater ROS levels and induction of PGC-1α transcription [106].

Li and collaborators demonstrated that SIRT1 activation by RVT resulted in the suppression of the STAT3 signaling pathway, leading to the reduced proliferation of HepG2 cells exposed to high glucose [107]. This beneficial property of RVT may be important in the development of new therapeutic strategies for the treatment of hepatocellular carcinoma, as the risk of developing this disease is greater in diabetics than non-diabetics [108]. Forkhead proteins (FOX) are a family of transcription factors that exert regulatory activity over the expression of genes involved in many cellular processes, including cell growth, proliferation, differentiation and longevity (RF). Frescas and collaborators reported that the activation of SIRT1 by RVT resulted in the nuclear translocation of FOXO1 in hepatocytes, promoted transcription of key genes involved in hepatic glucose production and, indeed, enhanced glucose release from the cultured hepatoma cells [109]. Conversely, Lin and co-workers demonstrated in rats that the activation of SIRT1 by RVT resulted in an opposite effect and blocked FOXO3 accumulation in the nuclei. This blocking effect may be important, as nuclear accumulation of FOXO3 can trigger downstream proteins that regulate inflammation and cell death [110]. Similarly, Bai and co-workers reported a reduction in FOXO1 expression in primary pig preadipocytes after RVT-induced SIRT1 activation. This inhibitory effect led to the significant inhibition of preadipocyte proliferation and differentiation into adipocytes [111].

### 3.2. Cardioprotection

RVT was found to be correlated with cardiac-specific overexpression of endogenous SIRT1 and its cardioprotective effects [112]. Doxorubicin (DOX) is an anthracycline antibiotic widely used to treat cancer with significant toxic side effects to healthy tissues, especially to the heart [113]. RVT plays an important role in preventing DOX-induced cardiotoxicity and the loss of heart function [114]. RVT administration in Balb/c mice at 15 mg/kg per day for seven weeks attenuated DOX-induced cardiotoxicity by upregulating SIRT1 [115]. Even in the aged heart, RVT countered the deleterious effects of DOX by restoring SIRT1 activity [116]. It has also been reported that RVT inhibited ROS and inhibited inflammation and pro-apoptosis in DOX-treated mice by enhancing SIRT1 and the p38MAPK pathway [117]. In vitro, RVT prevented DOX-induced cell death by downregulating endoplasmic reticulum (ER) stress in an SIRT1-dependent manner [118]. RVT treatment also activated SIRT1 and enhanced repair mechanisms in DOX-poisoned human cardiac progenitor cells (hCPCs). In hCPCs, the activation of SIRT1 regulates the expression of genes related to stress response, cell death and senescence by modulating several non-histone substrates such as p53 [119].

The regulation of acetylation/deacetylation of Foxo transcription factors by RVT via SIRT1 remains unresolved and may vary with different cell lines and subfamilies of Foxo [120,121,122]. It has been demonstrated that RVT plays a role in FoxO1-SIRT1 complex disassembly by decreasing the expression of FoxO1 in myoblast cells, whereas the SIRT1 inhibitor nicotinamide has been reported to upregulate FoxO1 [123]. Avogaro and co-workers have demonstrated a cardioprotective role for SIRT1 during ischemia-reperfusion (IR) injury as it increases the expression of FoxO1. They show that the translocation of FoxO1 protein from the cytoplasm to the nucleus is induced by IR. The cardioprotective effects of RVT derive from its antiapoptotic and antioxidant effects and due to its positive modulation of SIRT1 expression [124]. After a long-term treatment, RVT improved cardiac function by repressing the acetylation of Foxo1 in the senescent mouse heart [125].

The adverse vascular effects of cigarette smoke were also abrogated by RVT via a SIRT1-dependent pathway. The NF-κB activity was considerably decreased, and remarkable improvements in endothelial function were reported upon RVT pretreatment in Wistar rats [126]. The activation of the SIRT1-NF-κB pathway has also been reported to ameliorate subacute intestinal IR in BALB/c mice. RVT plays a role in this pathway by initiating protective events such as the regulation of inflammation by reducing NO production [127]. Evidently, hydrogen peroxide (H_2_O_2_) activates a precise intracellular signaling pathway responsible for NF-κB activation [128]. The overexpression of SIRT3 by RVT protected the H9c2 embryonic rat heart-derived cell line against H_2_O_2_-induced cell death via the NF-κB pathway, whereas the SIRT inhibitor enhanced cell death [123].

Mitochondria are the main source of intracellular ROS, and cardiovascular risk is related to excessive mitochondrial ROS (mtROS) [129]. Mn-SOD is an important intracellular anti-oxidative mechanism that helps balance ROS levels [130,131]. RVT treatment protects cardiomyocytes from oxidative stress by suppressing ROS production and upregulating mitochondrial genes (e.g., Mn-SOD) via the SIRT1 pathway. It has also been reported that its inhibitor (nicotinamide) downregulates Mn-SOD [132]. RVT also attenuated mtROS-mediated oxidative injury in human umbilical vein endothelial cells (HUVECs) via SIRT3 activation [133]. Xu and coworkers showed that the upregulation of Mn-SOD occurs via the extracellular-signal related kinase 5 (ERK5)/HDAC5 pathway, which then reduces mtROS levels in HUVECs [134].

The transcriptional co-activator p300 plays an essential role in cardiac development and also in cardiac hypertrophy [135]. Long-term RVT supplementation suppressed cardiac p300 upregulation and improved cardiomyopathy in dystrophin-deficient mdx mice. RVT also downregulates the p300 protein levels by activating SIRT1 [136].

During acute myocardial infarction (MI), stromal cell-derived factor (SDF)-1 and cardiac p53 levels are increased, and SDF-1 is known to recruit stem cells to the ischemic area [137]. RVT supplementation decreased the activity of p53 by deacetylation and increased SDF-1 levels at the injury site. In addition, a greater extent of the myocardium was found in the scar tissue four weeks after the acute MI in the RVT group, compared to the controls [138].

Severe hemorrhage triggers whole body hypoxia and induces oxidative injury upon reoxygenation [139]. The genomic studies have identified C-myc as one of the genes that are altered after a trauma-hemorrhage (T-H) event. C-myc was reported to be increased and linked to the downregulation of SIRT1 following such types of injury [140]. The data showed an inverse association between C-myc and SIRT1 expression [141]. Thus, RVT treatment activated SIRT1 and inhibited C-myc expression, leading to the deacetylation and inactivation of this gene as negative feedback [142]. The expression of pyruvate dehydrogenase kinase (PDK)-1 increased after a T-H event in male Sprague–Dawley rats. These high PDK-1 levels triggered a switch in cellular energy use from mitochondria-based to glycolysis. The administration of RVT increased SIRT1 activity and restored the glycolytic-mitochondrial energy pathway after a T-H event [143]. 

Adventitious fibroblasts have been reported to be involved in many cardiovascular events, including atherosclerotic plaques [144]. Activated adventitial fibroblasts can convert into myofibroblasts, synthesize collagen and enhance extracellular matrix remodeling and restructuring [145]. Cheng and coworkers have demonstrated an anti-atherosclerotic effect of RVT as it inhibits adventitial fibroblasts and the induction of cell apoptosis [146].

The structure and architecture of the heart undergo changes during cardiac fibrosis mediated by the cytokine, transforming growth factor (TGF-β), which has a potent collagen-producing function in cardiac fibroblasts [147]. Such profibrotic effects lead to increased TGF-β levels in the extracellular matrix [148]. Simultaneously, Smad proteins are activated by this cytokine and contribute to collagen deposition. Bu and coworkers showed that an RVT treatment leads to the activation of SIRT3 and the inhibition of the TGF-β/Smad3 pathway in mouse hearts. Specifically, it led to the inhibition of fibroblast to myoblast transformation and improving cardiac fibrosis and cardiac function [149].

### 3.3. Cancer

The potential antitumorigenic activity of RVT by affecting the HDAC pathway has been demonstrated in a number of tumor models, both in vitro and in vivo [150]. In prostate cancer, a phosphatase and tensin homolog (PTEN), a tumor suppressor, is deleted on chromosome 10 or inactivated by the negative regulator metastasis-associated protein 1 (MTA1)/HDACs. RVT targets the MTA1/HDAC complex by abrogating its negative epigenetic effect and may reactivate the tumor suppressor gene *PTEN* [151]. The downregulation of MTA1 by RVT disrupts the MTA1/HDAC complex, activating the proapoptotic genes Bax and p21 and triggering apoptosis in prostate cancer cell lines (PCa) [152]. In addition, it was reported that MTA1 downregulation by RVT is mediated through p53 acetylation in PCa cells [153]. Kumar and coworkers have reported that SIRT1 mediates the inhibition of phosphorylated ribosomal protein S6 kinase (pS6K) after RVT treatment in human prostate cancer cell lines. RVT also inhibited cell growth as it induced apoptosis through the inhibition of SIRT1-mediated S6K phosphorylation [154].

In colon cancer (CC), SIRT1 is downregulated and NF-κB increases, whereas RVT administration reverses this process [155]. The overexpression of SIRT1 showed an antiproliferative effect in CC cell lines [156]. In colorectal cancer (CRC) cell lines, RVT inhibited cell proliferation, invasion and metastasis by modulating cell cycle regulatory genes, enhancing apoptosis via upregulation of p53 and by inhibiting anti-apoptotic genes. Similar to CC, NF-κB was down-regulated in CRC cell lines after RVT treatment. This inhibition of NF-κB nuclear translocation was reversed when SIRT1 mRNA levels were suppressed, thus suggesting that RVT acts in an SIRT1-dependent mode [157]. Moreover, RVT downregulated SIRT1 and SIRT2 levels in human dermal fibroblasts, inducing premature senescence, which is associated with DNA damage [158].

Cozzi and co-workers demonstrated the potential use of RVT as a radioprotector in human lymphocytes. Their data suggested that RVT radiosensitizes non-proliferating human cells by modulating the acetylation status of histones (i.e., HDAC inhibition) [159]. In Hodgkin lymphoma cells, RVT enhanced the apoptosis by SIRT1 inhibition and p53/FOXO3a hyperacetylation. After RVT treatment, the levels of acetyl-p53 and acetyl-FOXO3a were increased, implying that deacetylase inhibition is a key factor in apoptosis of lymphoma cells due to RVT treatment [160].

The human T-cell leukemia virus type 1 (HTLV-1) can cause T-cell leukemia, and there are few therapeutic options available for these patients [161]. HTVL-1 is responsible for encoding the Tax oncoprotein, which activates numerous cellular promoters [162]. The suppression of Tax activation by HTVL is carried out by SIRT1 deacetylase. The administration of RVT activated SIRT1 in HTLV-1-transformed T cells, which inhibited HTVL-1 proviral transcription and Tax expression [163].

RVT treatment in hepatoma cell lines suggested dose-dependent antiproliferative effects. In HepG2 cells, an HDAC inhibition assay revealed that this effect occurs because of the specific inhibition of certain HDAC families and hyperacetylation of the histone proteins [164]. Lindgren and coworkers also revealed potential antiproliferative effects of RVT. Their group carried out a study to evaluate the effect of RVT on growth and apoptosis promotion in osteoblastoma cell lines. After RVT treatment, the SIRT1 protein was overexpressed in osteosarcoma cells compared to normal osteoblasts. Additionally, RVT induced apoptosis in a dose-dependent manner and had minimal effects on normal osteoblasts [165]. RVT can also attenuate androgen receptor-mediated proliferation on breast cancer cells by activating SIRT1 [166].

### 3.4. Neuroprotection

Alzheimer’s disease (AD) is a neurodegenerative disorder characterized by progressive memory deficit, cognitive impairment and personality changes. Typically, AD is manifested as extracellular deposits of amyloid-β (Aβ) in the form of senile plaques in the brain. Aβ also deposits in the cerebral blood vessels and intracellular inclusions of hyperphosphorylated tau in the form of neurofibrillary tangles (NFT) [167,168]. It is hypothesized that the pathogenic engagement of microglia with Aβ involves the activation of NF-κB. The RelA/p65 in AD brains is greater in neurons and astrocytes than that surrounding amyloid plaques, demonstrating a role for NFkB in AD pathogenesis [169]. A complete transcriptional activity of RelA/p65 requires acetylation of Lys310, a known target of SIRT1 deacetylase [170]. Chen and co-workers were able to demonstrate the ability of RVT to protect mixed cortical cultures against Aβ toxicity as it inhibits microglial NF-κB signaling by activating SIRT1 [171].

In a different perspective, researchers have studied a possible role for the ROCK protein in mediating the protective effects of RVT in AD. ROCK is a serine/threonine protein kinase with two different diastereomers: ROCK1 and ROCK2. ROCK1 is known for partly inhibiting the non-amyloidogenic α-secretase processing of the amyloid precursor protein (APP), which is suggested to be partly modulated by SIRT1 overexpression [172,173]. Based on these data, the ability of RVT to increase cell viability and to inhibit cell apoptosis against Aβ_25–35_ toxicity has been demonstrated. Furthermore, it has been demonstrated that Aβ_25–35_ could decrease the SIRT1 expression and increase the ROCK1 expression, which is reversed by RVT treatment. Nicotinamide and Y27632, SIRT1 and ROCK1 inhibitors, respectively, were used to demonstrate the relation between SIRT1 and ROCK1 expression, and the results demonstrated that ROCK1 is a downstream molecule. Taken together, this study demonstrated the SIRT1-ROCK1 pathway to be partially responsible for RVT neuroprotection [174].

Akt, a serine/threonine kinase, plays a role in regulating neuronal cell size and survival, accelerating axonal regeneration and promoting axon elongation and branching [175,176,177,178]. Akt is also a downstream target of SIRT1 and can be modulated by this enzyme through deacetylation [179,180]. Based on this, Zhang et al. studied the role of the SIRT1-Akt pathway in RVT-mediated neuroprotection in Alzheimer disease. The results of their work demonstrated that RVT can increase the neuron viability and prevent neuron apoptosis when exposed to Aβ_25–35_. These cells showed a reduced expression of SIRT1 and Akt due to the Aβ_25–35_ treatment, which was reversed by RVT treatment. Nicotinamide was also able to partially mitigate RVT-mediated neuroprotection, suggesting that RVT could protect cortical neurons from Aβ_25–35_-induced damage, at least partly via the SIRT1-Akt1 pathway [181].

Parkinson’s disease (PD) is another common neurodegenerative disorder that is characterized by dopaminergic cell death in the substantia nigra. The earlier studies have demonstrated that neurotoxic chemicals are responsible for sporadic cases of PD [182,183]. These neurotoxins induce oxidative stress by elevating ROS concentrations, which are already relatively high in the dopaminergic neurons due to dopamine metabolism and auto-oxidation [184]. Methionine sulfoxide reductase A (MsrA) belongs to a catalytic antioxidant system, and its overexpression reduces the dopaminergic cell death and protein aggregation caused by the PD-related neurotoxins [185,186]. In a study that assessed the involvement of MsrA, SIRT1 and RVT in PD, Wu and collaborators demonstrated that phytoalexin was able to enhance the resistance of human neuroblastoma SH-SY5Y cells to 1-methyl-4-phenylpyridinium ion (MPP^+^)-induced neurotoxicity and that it upregulates MsrA expression. Nicotinamide, an SIRT1 inhibitor, was able to counter this increase in MsrA expression, suggesting that SIRT1 is involved in this process [187].

Another important aspect pertains to the sustained activation of the glia (macroglia and microglia) in the CNS, which is believed to contribute to progressive dopaminergic (DA) neurodegeneration in PD via neuroinflammation [188]. Following an injury or infection, the activated microglia release various proinflammatory and other neurotoxic factors, the majority of which are deemed to contribute to neuronal injury. Ye and co-workers studied the role of SIRT1 in the neuroinflammatory process associated with PD. The results of their study revealed that RVT increases SIRT1 content in PC12 cells and suppresses the LPS-induced increase of IL-6 and TNF-α. In addition, sirtinol was able to abolish this RVT effect, demonstrating the involvement of SIRT1 in regulating proinflammatory cytokine release after microglial activation [189].

Axonal degeneration is common in many neurodegenerative diseases and is particularly important in the development of amyotrophic lateral sclerosis, Charcot-Marie-Tooth disease, spinal muscular atrophy and diabetic neuropathy [190]. Calliari and collaborators, using a Wallerian degeneration model, studied the role of SIRT1 and SIRT2 in the modulation of the axonal degeneration, when cells were treated with RVT. In the first case, it was demonstrated that RVT delayed axonal degeneration, but this was lost by the addition of suramin, an SIRT1 inhibitor. Moreover, RVT was able to dissociate DBC1 (Deleted in Breast Cancer-1, an endogenous SIRT1 inhibitor) from the SIRT1 catalytic site in neurons, which then increased enzyme activity [191]. On the other hand, Suzuki and coworkers proved that RVT decreases the resistance of Wlds neurons to axonal degeneration induced by colchicine and that it also decreases the level of tubulin acetylation. The deacetylation of tubulins has been suggested to be involved in this process due to the SIRT2 activation, and resistance to the axonal degeneration was restored when SIRT2 was silenced [192].

Another important neurodegenerative disease, amyotrophic lateral sclerosis (ALS), is characterized by motoneurons’ (MN) death, which results in the progressive muscle atrophy and paralysis [193]. An interesting finding in ALS was the identification of mutations in the gene encoding SOD1 (superoxide dismutase 1), which were observed in 20% of inherited ALS cases [194]. In a study using transgenic mice that harbored the G93A human SOD1 mutation, Mancuso and collaborators showed the ability of RVT in delaying the onset of symptoms and in improving impaired locomotion associated with spinal motoneuron degeneration. Moreover, the RVT treatment also increased the expression and activity of SIRT1 in motoneurons, suggesting a possible mechanism of action for RVT in easing the symptoms of ALS [195].

With the aim of studying the role of SIRT1 in ALS, Wang and collaborators used the motor neuron-like cells with the hSOD1 G93A mutant vector (VSC-4.1-hSOD1^G93A^) for their experiments and observed both low expression of SIRT1 and lower ATP levels. RVT treatment was able to increase the viability of VSC-4.1-hSOD1^G93A^ cells, SIRT1 expression and ATP levels. Nicotinamide, an SIRT1 inhibitor, was able to reduce these effects, suggesting that SIRT1 is involved in ALS [196]. 

Pérez-Pinzón and co-workers studied the efficacy of ischemia pre-conditioning (IPC) and RVT treatment against cerebral ischemia. In two different reports, they were able to demonstrate that RVT could mimic neuroprotection achieved with IPC and that both were able to induce tolerance against brain injury and increase SIRT1 activity. Sirtinol, an SIRT1 inhibitor, was able to abolish the neuroprotective effects of RVT and IPC, suggesting an important role for SIRT1. In a more recent report, they studied the involvement of UCP2 (mitochondrial uncoupling protein 2) in neuroprotection and showed that RVT treatment downregulates UCP2, which is important for neuroprotection, and they concluded that this protein protects against lethal ischemia, at least partly by increasing mitochondrial ATP production [197,198].

Further, Shin and collaborators established that RVT treatment significantly reduced total infarct volume in mice after ischemic stroke. They also demonstrated that RVT was able to increase SIRT1 expression and that this was correlated with PGC1α levels, a regulator of mitochondrial biogenesis and oxidative stress, suggesting that this process underlies the anti-oxidative neuroprotection associated with RVT in the ischemic brain [199].

Neuropathic pain symptoms can be related to alterations in the expression of genes associated with pain and modifications in the primary afferent or spinal cord neurons. Abnormal histone acetylation is one of the characteristic gene modifications and is believed to be one of the transcription factor-mediated epigenetic mechanisms that underlie neuropathic pain [200,201]. With an aim to study the role of SIRT1 and RVT in neuropathic pain, Shao and co-workers performed an in vivo study in a mouse model of chronic constriction injury (CCI). They observed that CCI surgery decreased SIRT1 expression, activity and the NAD/NAM ratio. RVT (90 mM) treatment was able to delay the development of neuropathic pain induced by CCI and increased SIRT1 activity. The treatment with 6-chloro-2,3,4,9-tetrahydro-1*H*-carbazole-1-carboxamide (EX527), an SIRT1 inhibitor, blocked the anti-nociceptive effect of RVT. The results established a possible role of SIRT1 in neuropathic pain and suggested that increasing its activity could be a promising approach to treat neuropathic pain [202].

Regarding neuronal death, it was assessed the ability of RVT and SIRT1 to increase neuronal cell viability upon induction of apoptosis. Seo and collaborators used PrP (106–126), a synthetic human prion peptide, to induce cell death in the human neuroblastoma cell line SH-SY5Y. PrP, besides reducing cell viability, also inactivated SIRT1. RVT treatment significantly inhibited PrP (106–126)-mediated neuronal cell death and increased SIRT1 activity and expression. Sirtinol, as expected, blocked an RVT-mediated increase in SIRT1 protein activity and increased PrP (106–126)-induced cell death [203]. Guida and co-workers assessed the behavior and expression of the *REST* gene (repressor element 1 (RE-1)-silencing transcription factor) in RVT-treated cells. REST is known to be involved in neuronal death in cortical neurons. These authors observed that RVT reduced REST mRNA and protein expression in a dose-dependent manner. In order to evaluate the role of SIRT1 in RVT-induced changes in REST expression, EX527 was used, and it leads to an increase in *REST* gene expression and blocked the effects of RVT. Polychlorinated biphenyl-95 (PCB-95) was used to induce neuronal death in SH-SY5Y cells, which could be reversed by RVT treatment and also by decreasing *REST* gene expression [204].

Proinflammatory changes may induce cognitive deficits in rodents and the injections of LPS into the CA1 region of the hippocampus cause learning and memory deficits in mice [205,206]. Thus, the role of RVT on LPS-induced dysfunction and cognitive memory loss in mice was analyzed. An LPS treatment decreased SIRT1 protein, but the RVT treatment was able to reverse it and lessen the memory deficit. A specific SIRT1 inhibitor abolished the neuroprotective effects of RVT, suggesting that the mechanism of action involved activation of SIRT1 [207].

Morphine is a powerful analgesic used for treating severe pain, but induces tolerance when used in the long term. Recent studies have suggested that the expression of genes related to pain is involved in the generation and maintenance of neuropathic pain and morphine tolerance [208,209]. A study determining the effects of RVT treatment in rats exhibiting morphine tolerance showed that RVT was able to reverse a morphine-dependent increase in pro-inflammatory cytokines and increased the analgesic effects of morphine in tolerant rats. Moreover, the authors also suggested that morphine tolerance was associated with a significant time-dependent increase in HDAC-1 protein levels, which was reversed by RVT [210].

Proximal spinal muscular atrophy (SMA) is a neuromuscular disorder caused by the degeneration of alpha motor neurons and is characterized by a mutation in the survival motor neuron gene (*SMN1*); however, all patients retain a nearly identical copy of SMN2 [211,212]. With the aim of developing a strategy to increase SMN2 expression, Dayangac-Erden and co-workers studied the ability of RVT to inhibit HDAC activity and change SMN2 expression. The results revealed that (*E*)-resveratrol inhibited HDAC activity in a dose-dependent manner and that it was able to increase SMN2 mRNA and protein levels in 3813 cell lines. Therefore, the authors concluded that HDAC inhibition may be a possible strategy for treating proximal spinal muscular atrophy [213].

### 3.5. Inflammation

The control of inflammatory processes by sirtuins is still being studied, and its mechanism is not completely known; however, it is associated with histone changes and transcription factors such as NF-κB and AP1 [214]. The existing evidence suggests that during chronic inflammation, NAD^+^ and SIRT levels, as well as SIRT expression are drastically reduced, which may lead to an increase in NF-κB RelA/p65 activity, thus increasing the expression of pro-inflammatory cytokines [215].

Osteoarthritis and rheumatoid arthritis are both joint disorders characterized by excessive production of inflammatory mediators synthesized by local synovial fibroblasts and chondrocytes [216]. To demonstrate the role of SIRT1 in inflammation related to osteoarthritis, Moon and collaborators used human articular chondrocyte cell culture and TNF-α-induced inflammation. RVT treatment was able to prevent COX-2 and MMP mRNA expression in a dose-dependent manner and was also able to inhibit COX-2 protein expression. RVT also was able to induce the expression of SIRT1 in these cells. Further, the anti-inflammatory effect of SIRT1 could be related to NF-κB activity as RVT was able to decrease acetylation of NF-κB-p65 [217]. Similar results regarding COX-2 and SIRT1 expression have been reported in a study conducted by Yang and co-workers, who used bradykinin to induce inflammation in human rheumatoid arthritis synovial fibroblasts (RASFs). In addition, RVT treatment can also decrease prostaglandin 2 production and reduce the activity and acetylation of the transcription factors NF-κB and AP-1, which regulate the production of pro-inflammatory cytokines [218]. 

Inflammatory bowel diseases (IBD), such as ulcerative colitis and Crohn’s disease, are chronic relapsing inflammatory conditions of the gastrointestinal tract that result in tissue destruction and mucosal injury in the colon [219]. Sharma and collaborators evaluated the effects of RVT in IBD in an in vivo study using the dextran sulfate sodium (DSS)-induced colitis model. The treatment with RVT was able to reduce tissue levels of TNF-α, IFN-γ and IL-17, obtained by biopsy. An increase in SIRT1 mRNA expression was also observed. In addition, an increase in the tissue inhibitor of metalloproteinase (TIMP)-3 mRNA and a decrease in TNF-a converting enzyme (TACE) mRNA was observed [220].

Knobloch and co-workers evaluated the effects of RVT and corticosteroids (dexamethasone) on human airway smooth muscle cells (HASMCs) after exposure to (lipoteichoic acid) LTA. It is known that these cells play a role in airway inflammation in chronic obstructive pulmonary disease (COPD) and release cytokines and chemokines in the presence of bacterial pathogen associated molecular patterns (PAMPs), leading to exacerbation of the disease and irreversibly accelerate disease progression [221,222]. The results of this study showed that both RVT and dexamethasone were able to reduce monocyte chemotactic protein (MCP-1/CCL2) and interleukin-6 (IL-6) levels in a concentration-dependent manner, but RVT was more efficient in exerting the same effect. Both compounds also reduced the levels of granulocyte-macrophage colony-stimulating factor (GM-CSF) released from HASMCs after an exposure to LTA [223]. 

Pan and co-workers studied the molecular basis of the anti-inflammatory effect of RVT on human umbilical vein endothelial cells (HUVEC) after TNF-α-induced damage. TNF-α decreased the cell viability and induced the expression of CD40. RVT was able to reverse this decrease in cell viability and reduce CD40 expression. It has also been observed that TNF-α inhibits the production of SIRT1, whereas the treatment with RVT increased SIRT1 expression. To assess whether the RVT-induced reduction in CD40 expression is linked to its ability to increase SIRT1 expression, the authors used knocked-out SIRT1 and used an SIRT1 inhibitor, both of which led to the suppression of the beneficial effects of RVT. It was also observed that the RVT treatment was able to suppress NF-κB activity [224].

Sepsis, an inflammatory response to a severe infection, is mediated by several types of immune cells and involves the production of pro-inflammatory cytokines. In normal conditions, a moderate production of cytokines facilitates limiting infections and tissue damage. As the infection terminates, the inflammatory response also ends, and the organism returns to homeostasis. The production of high levels of TNF-α is considered important for the development of sepsis [225,226,227]. To assess whether RVT may have some benefits in sepsis, Chen and coworkers used an in vitro model with normal and LPS-tolerant THP-1 cells to induce an inflammatory response. The results showed that pre-conditioning with RVT was able to decrease TNF-α levels by activating SIRT1 in normal and LPS-tolerant human monocytic cells (THP-1), which then led to the repression of TNF-α transcription through deacetylation of H4K16 in the TNF-α promoter. In addition, it was also observed that RVT does not increase the levels of the SIRT1 protein in tolerant cells, but only in normal cells. According to these authors, this effect indicates that, in tolerant cells, SIRT1 levels reached their maximum, and any influence of RVT remained insignificant [228].

An exposure to ionizing radiations can induce an increase in ROS and IL-1β levels, but the mechanism(s) involved remains unclear. NLRP3 is the most-studied inflammasome and can be activated by pathogens, damage to molecular patterns or environmental conditions. When activated, this inflammasome is capable of inducing the conversion of pro-IL-1β to its activated form. Although the mechanism by which NLRP3 is activated is not completely understood, it is known to require NF-κB activation [229,230]. To understand if the radiation-induced pro-inflammatory activity is related to the activation of the NLRP3 inflammasome and a subsequent increase in IL-1β, Fu and co-workers evaluated the effects of radiation on mesenchymal stem cell (MSCs) cultures. After an exposure to radiation, MSCs showed elevated levels of IL-1β, and the RVT treatment resulted in a concentration-dependent decrease in the expression of IL-1β and NLRP3, as well as an increase in the SIRT1 protein expression. To confirm the participation of SIRT1 in these events, the same experiments were repeated using a known SIRT1 inhibitor, namely nicotinamide. As expected, the pre-treatment with NAM decreased the levels of SIRT1 and increased the levels of NLRP3 and IL-1β. Similar results were obtained with SIRT1 knockdowns [231].

Another study evaluated if RVT treatment can diminish the effects of ionizing radiation in vivo after radiotherapy-induced premature ovarian failure (POF). These authors also analyzed certain inflammatory aspects by immunohistochemistry and real-time PCR. Radiation exposure led to elevated levels of IL-6 and IL-8 (pro-inflammatory) mRNAs and decreased levels of IL-10 (anti-inflammatory) mRNA. Immunohistochemistry showed an increase in NF-κB-p65. The use of RVT led to a decrease in the levels of pro-inflammatory interleukins, but an increase in IL-10 levels, reduction in NF-κB-p65 levels in analyzed tissues and an increase in SIRT1 mRNA [232].

### 3.6. Lifespan

In a study on lifespan changes due to small molecule activators of sirtuins, Howitz and collaborators demonstrated that RVT was able to increase the lifespan of *Saccharomyces cerevisiae* by up to 70%, and cell survival in human osteosarcoma (U2OS) and human embryonic kidney (HEK 293) cells. The authors also report that RVT increased the rate of deacetylation of *SIRT1* by two-fold and decreased the acetylation of the p53 gene, an *SIRT1* target, suggesting that *SIRT1* activation could be a possible mechanism that increases lifespan upon RVT treatment. Moreover, the Sir-2 protein family is known to extend yeast lifespan by stabilizing repetitive DNA [233,234,235,236], which reach toxic levels in old cells and cause cell death. RVT was also able to reduce the frequency of rDNA by about 60% in an SIR2-dependent manner [237].

With the aim of studying lifespan in multicellular animals, Wood and collaborators used *Caenorhabditis elegans* and *Drosophila melanogaster* as experimental models. They were able to demonstrate that RVT increased deacetylation up to 2.5-fold for Sir-2.1 of *C. elegans* and 2.4-fold for Sir-2 of *D. melanogaster*. Moreover, the lifespan of the worms was extended by 14% while that of *D. melanogaster* increased by 29%. These results indicate that the effects of RVT on lifespan are dependent on Sir-2, and RVT did not change the fecundity of the animals [238]. In another study on *C. elegans* lifespan, Viswanathan and co-workers also demonstrated that RVT can extend lifespan in a dose-dependent manner that is completely dependent on Sir-2.1. In addition, these authors also discovered that this effect was independent of daf-16, an insulin/IGF pathway transcription factor that has been shown to regulate nematode aging. Additionally, polyphenols have also been shown to extend *C. elegans* lifespan by inhibiting Sir-2.1-mediated repression of ER stress genes, which are determinants of lifespan [239].

Recently, it has been demonstrated that autophagy is responsible for increasing longevity through a cytoprotective mechanism that allows cells to mobilize their energy reserves and to recycle damaged organelles [240,241,242,243]. Specifically, Morselli and co-workers have studied the role of RVT and SIRT1 in autophagy and lifespan. Their results revealed that RVT and caloric restriction stimulate autophagy through SIRT1 activation both in human cells and *C. elegans* and that SIRT1 knockout nullified this effect, which implicates a role for SIRT1 and RVT in autophagy and lifespan expansion [244].

In addition, RVT has also been demonstrated to extend lifespan of more complex organisms such as *Nothobranchius guentheri*, the annual fish. The phytoalexin was able to prolong fish lifespan and retarded the expression of aging-related biomarkers [245]. On the other hand, the ability of RVT to extend lifespan in mice is not well stablished. While studies have demonstrated that RVT is not able to extend lifespan in healthy mice [246,247], supplementation of metabolically-compromised mammals has demonstrated to be successful [248].

## 4. Lysine-Specific Demethylase-1

Lysine-specific demethylase-1 (LSD1), an enzyme that regulates histone methylation, belongs to the superfamily of the flavin adenine dinucleotide (FAD)-dependent amine oxidases [249]. The catalytic function of LSD1 involves the removal of mono- or di-methyl groups from methylated proteins, specifically histone 3, lysine 4 and 9 (H3K4 and H3K9) [250,251]. The demethylation of H3K4 results in the repression of gene expression [251], while demethylation in H3K9 leads to activation of gene expression [252,253]. The epigenetic reprogramming modulated by LSD1 plays an important role in a number of normal physiological processes, including cell growth, differentiation and metabolism [254]. Nevertheless, earlier studies have related the overexpression of LSD1 with a variety of diseases, including cancers, neurodegenerative diseases and hematological disorders [255,256,257,258,259]. In view of this promising epigenetic target, Yang and coworkers assessed the effects of several natural polyphenols against LSD1, including RVT, curcumin, quercetin and luteolin. Interestingly, RVT was able to inhibit LSD1 activity in a dose-dependent manner with an IC_50_ of 15 µM using an in vitro system of recombinant LSD1 enzyme and a short peptide of methylated p53 as the substrate. Furthermore, these authors also performed a study using purified bulky histones as substrates, and RVT could inhibit LSD1 activity at the histone lysine 4 site [260].

## 5. Conclusions

RVT is a natural polyphenol with promising therapeutic applications in a variety of diseases, including cancer, diabetes, cardiovascular diseases, neurodegenerative diseases and metabolic disorders. Both in vitro and in vivo data demonstrate the potential effects of this polyphenol in treating and preventing many of these diseases. Specifically, RVT affects epigenetic mechanisms, which include enzymes such as DNA methyltransferase (DNMT), HDAC and lysine-specific demethylase-1 (LSD1). The known effects of RVT on these enzymes, specifically against HDAC, are well established and are responsible for several of the observed beneficial effects.
